
JOURNAL INFORMATION
==============================
NLM Title Abbreviation: ScientificWorldJournal
Journal ID: ScientificWorldJournal
NLM Journal ID: 101131163
Journal ID: TSWJ

The Scientific World Journal

ISSN: 2356-6140
EISSN: 1537-744X
Publisher: Wiley

ARTICLE INFORMATION
==============================
PMCID: PMC6084227
DOI: 10.1100/tsw.2001.403
Article ID: 561279
Article version: 1
Subjects: Extended Abstract

Modulation of the Expression of Calcitonin Gene-Related Peptide and Substance P in Cultured Dorsal Root Ganglia by Opioid Receptor Agonists. Involvement of the PKC Pathway

Bolanger S. 1 2
Ma W. 2
Chabot J.-G. 2
Quirion R. 1 2
1 Department of Pharmacology and Therapeutics, McGill University, Verdun, QC, Canada H4H 1R3,
2 Douglas Hospital Research Center and Department of Psychiatry, McGill University, Verdun, QC, Canada H4H 1R3,
Publication date: 2001
Electronic publication date: 2001 Oct 18
Volume: 1
First page: 36
Last page: 36
Copyright: Copyright © 2001 S. Bolanger et al.
Copyright year: 2001
License: This is an open access article distributed under the Creative Commons Attribution License, which permits unrestricted use, distribution, and reproduction in any medium, provided the original work is properly cited.
License URL: https://creativecommons.org/licenses/by/3.0/

Funding: Canadian Institutes of Health Research

==============================
4th International Meeting on Calcitonin Gene-Related Peptide (CGRP)
*TheScientificWorld* (2001) 1(S1), 36
ISSN 1532-2246; DOI 10.1100/tsw.2001.403

### MODULATION OF THE EXPRESSION OF CALCITONIN GENE-RELATED PEPTIDE AND SUBSTANCE P IN CULTURED DORSAL ROOT GANGLIA BY OPIOID RECEPTOR AGONISTS. INVOLVEMENT OF THE PKC PATHWAY

S. Bélanger[1,2], W. Ma[2], J.-G. Chabot[2], and R. Quirion[1,2]
[1]Department of Pharmacology and Therapeutics, [2]Douglas Hospital Research Center and Department of Psychiatry, McGill University, Verdun, QC, Canada, H4H 1R3

The mechanisms involved in morphine tolerance are poorly understood. It was reported that calcitonin gene-related peptide (CGRP) immunoreactivity was increased in the spinal dorsal horn during morphine tolerance and an *in vitro* repetitive morphine treatment was able to mimic the *in vivo* results by inducing increases in CGRP- and substance P (SP)-immunoreactive (IR) neurons in cultured dorsal root ganglia (DRG). The aim of this study was to establish which subtypes of opioid receptors are involved in the induction of CGRP and SP in cultured rat DRG neurons and to examine the signaling pathway involved in the induction of these neuropeptides following repetitive opiate treatments.

Following treatment with either of the three opioid agonists [μ; DAMGO, δ; DPDPE, κ; U50488H], the number of CGRP- and SP-IR neurons increased significantly, in a concentration-dependent manner. Double-immunofluorescence staining showed that the three opioid receptors were colocalized with both of the pain-related neuropeptides. Protein kinase C (PKC)-IR was found to be significantly increased following a repetitive treatment with DAMGO. Double-immunofluorescence staining showed the colocalization of PKCα with CGRP or SP in cultured DRG neurons. Moreover, a combined treatment of opioids with PKC inhibitors (chelerythrine chloride or GO6976) was able to block the effects of the opioid treatment on increased CGRP-like IR.

These data suggest that the three opioid receptors are expressed in DRG neurons and may be involved in the induction of CGRP and SP by opiates and that PKC probably plays a role in the signaling pathway leading to the upregulation.

Supported by grant from CIHR.
